# The Role of Groove Periodicity in the Formation of Site-Controlled Quantum Dot Chains

**DOI:** 10.1186/s11671-015-0938-8

**Published:** 2015-05-28

**Authors:** Andreas Schramm, Teemu V Hakkarainen, Juha Tommila, Mircea Guina

**Affiliations:** Optoelectronics Research Centre, Tampere University of Technology, P.O. Box 692, FIN-33101 Tampere, Finland

**Keywords:** III-V semiconductors, InAs Quantum dots, Site-controlled quantum dots, Molecular beam epitaxy, Nanoimprint lithography

## Abstract

Structural and optical properties of InAs quantum dot (QD) chains formed in etched GaAs grooves having different periods from 200 to 2000 nm in [010] orientation are reported. The site-controlled QDs were fabricated by molecular beam epitaxy on soft UV-nanoimprint lithography-patterned GaAs(001) surfaces. Increasing the groove periods decreases the overall QD density but increases the QD size and the linear density along the groove direction. The effect of the increased QD size with larger periods is reflected in ensemble photoluminescence measurements as redshift of the QD emission. Furthermore, we demonstrate the photoluminescence emission from single QD chains.

## Background

Site-controlled quantum dots (QDs) have attracted great attention during the last decade due to their potential as novel applications in quantum information manipulation [1–3]. A requirement for these applications, for example, in demonstrating single [4, 5] or entangled photon sources [6], is the exact positioning of QDs on predefined locations allowing their addressing. In molecular beam epitaxy (MBE) processes, this can be achieved by defining the nucleation sites for impinging atoms using patterning the surface. Patterning is usually accomplished by lithographic techniques, such e-beam lithography [7–11], nanoimprint lithography [12], interference lithography, photolithography [13], or atomic force microscopy (AFM) lithography [14].

In recent years, site-controlled QD epitaxy was focused on creating periodic arrays of single QDs used, e.g., as single photon sources embedded in microcavities [15, 16]. Moreover, another interesting topic emerged related to ordering of QDs in various kinds of arrays, such as in chains of QDs. QD chains create a bridge from zero- to one-dimensional nanostructures and show interesting optical [17–19] and transport behaviors [20]. Previously, we could show that InAs QD chains grown onto groove-like patterns show different growing behaviors than their self-assembled counterparts, and thus their optical properties show differences [21]. These studies were focused on dense QD chains with a period of 200 nm and thus, we could mainly investigate the ensemble properties.

In this paper we are studying MBE-grown InAs QDs deposited on groove-patterned GaAs(100) that have periods of 500–2000 nm, enabling optical accessing of single QD chains. Patterning was performed by UV-NIL in different orientations on the substrate. We study the growth and optical properties and demonstrate μ-photoluminescence (μ-PL) measurements of single QD chains in [010] direction.

## Methods

Site-controlled InAs QDs were fabricated by MBE. First, a 500-nm GaAs buffer layer was grown on a GaAs(001) substrate. Second, soft UV-NIL was applied to pattern the GaAs surface. An optimized dry etching process was done to form grooves for QDs. Grooves in [010] orientation with a depth of 30 nm and periods of 200, 500, 1000, and 2000 nm were prepared on the same wafer. After the patterning, chemical cleaning and native oxide removal were performed, and the samples were loaded into the MBE chamber. The patterning and cleaning processes are described in more detail in [22]. Finally, a 60-nm GaAs buffer layer and 1.8 ML of InAs were deposited at 470 and 540 °C, respectively. For surface measurements, the sample was unloaded from the MBE chamber after the QD deposition. For PL studies, the QDs were capped by 20 and 50 nm of GaAs grown at 540 and at 590 °C, respectively. AFM and scanning electron microscopy (SEM) were used to study the surface samples. For ensemble PL experiments, the samples were loaded into closed-cycle cryostat, excited with a 488-nm laser, and measured with a PMT detector. For μ-PL measurements, the sample was loaded into a low-vibration closed-cycle helium cryostat and cooled down to 5 K. Nonresonant optical excitation at 532 nm was used, and the laser beam was focused on the sample with a ×50 high NA objective. The spot diameter onto the sample was approximately 1 μm. For intensity images, a diverging lens was used to increase the diameter of the laser spot on the sample to 30 μm. The emitted signal was collected by the same objective and dispersed with a 0.75-m monochromator equipped with a 1200 lines/mm grating and a cooled Si CCD camera.

## Results and Discussion

**Fig. 1:**
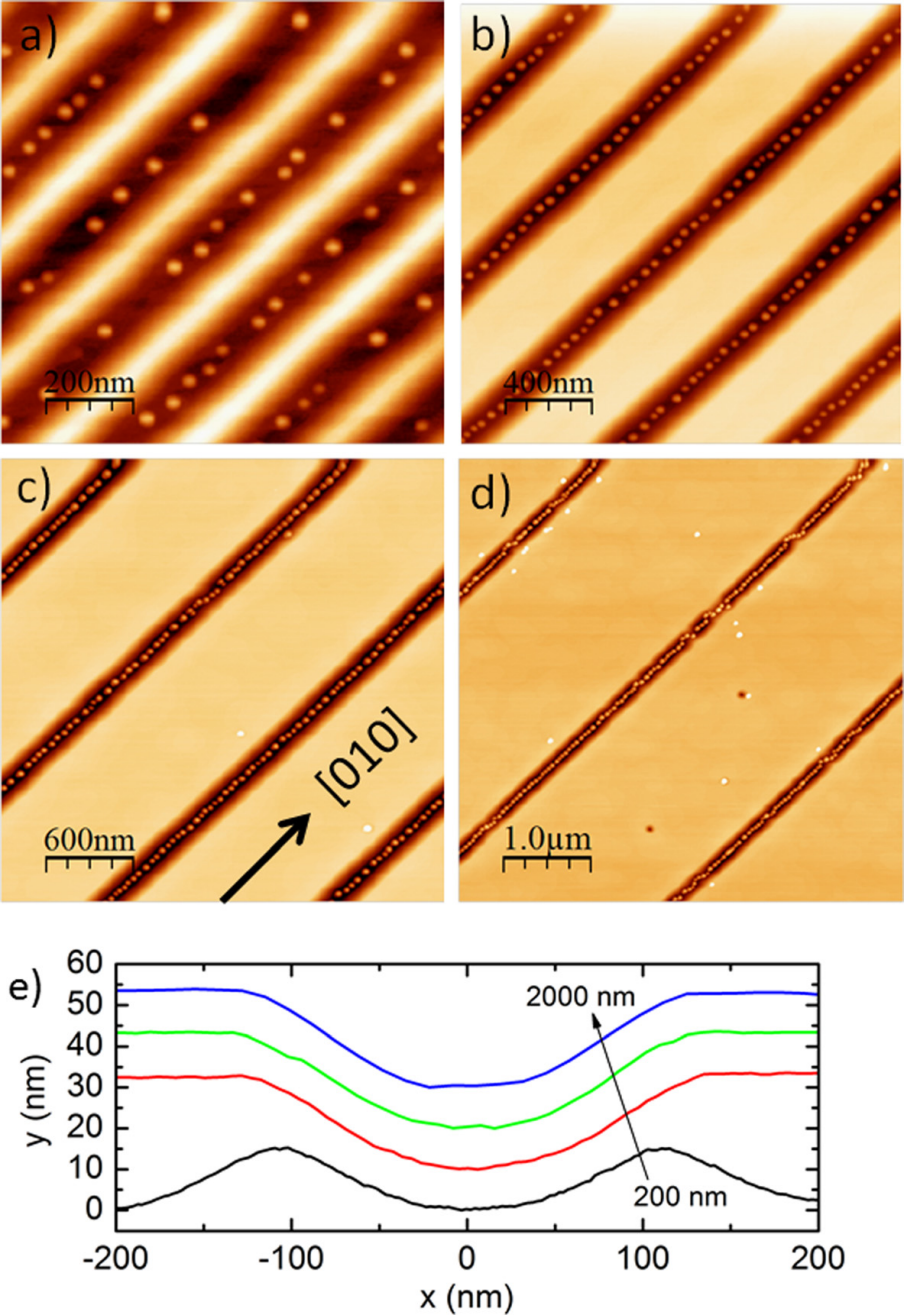
**a** 1, **b** 2, **c** 3, and **d** 5 μm^2^ AFM images of surface QDs with groove periods of 200, 500, 1000, and 2000 nm, respectively. **e** The corresponding line scans across the grooves

In Fig. 1a–d we show the AFM images of site-controlled surface QDs aligned in [010]-oriented grooves with periods of 200, 500, 1000, and 2000 nm, respectively. We used here in this experiment the [010] direction in order to circumvent any GaAs growth anisotropies we have observed previously [21]. We observe that nearly all the QDs have grown into the groove structures. Whereas in Fig. 1a, the 200-nm separated grooves are sparsely populated by QDs, and the QD density increases in the grooves with larger periodicity. The dot-to-dot distance decreases in the 500, 1000, and 2000-nm separated grooves and the QDs form QD chains. In Fig. 1d few parasitic QDs are observed between the grooves. The corresponding line scans of the grooves are presented in Fig. 1e which shows that the curvature of the grooves and the angle of the sidewalls are not depending on the pattern period.

**Fig. 2:**
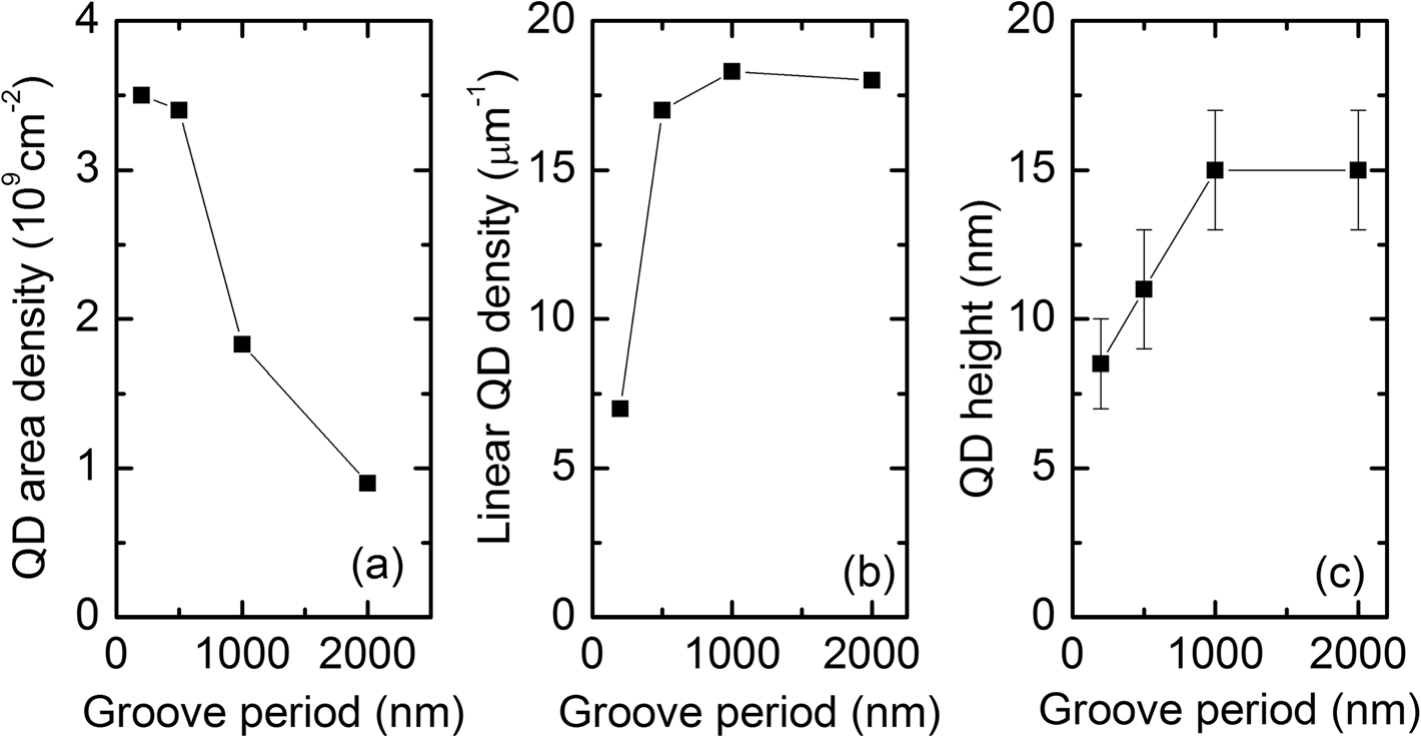
**a** QD density versus period of the grooves. **b** Linear QD density per one groove. **c** QD height versus groove period

The QD densities and average QD heights for the samples shown in Fig. 1a–d are summarized in Fig. 2. The overall QD area density decreases with increasing groove period from 3.5 × 10^9^ cm^−2^ to 0.9 × 10^9^ cm^−2^, as shown in Fig. 2a. According to Fig. 2b, the linear QD density along the groove direction increases from 7 μm^−1^ to around 18 μm^−1^ when the groove period is increased from 200 nm to 1000 nm and saturates at that level. At the same time, the QD height (Fig. 2c) increases with increasing groove period from 8.5 nm for 200-nm period to 15 nm for 1000-nm period and saturates at that level.

Typically, the formation of InAs QDs on a patterned surface is governed by migration of In adatoms on favorable sites. The adatom migration on a surface with nonuniform properties is driven by local gradients in the chemical potential, which for In can be expressed as [23, 24]

1 $$ {\mu}^{\mathrm{In}}\left(\mathbf{r}\right)=\left[{\mu}_0^{\mathrm{In}}+\frac{Y{\varepsilon}_{\left|\right|}^2\left(\mathbf{r}\right)\varOmega }{2}\right]+\gamma \varOmega \kappa \left(\mathbf{r}\right), $$

where ${\mu}_0^{\mathrm{In}}$ is the chemical potential of In on a flat, uniform reference surface. The second term is elastic correction to ${\mu}_0^{\mathrm{In}}$, where *Y* is Young’s modulus, *ε*_||_(**r**) the local in-plane elastic strain, and Ω the atomic volume. The third term is the surface energy contribution to the chemical potential, where *γ* is the surface energy and *κ*(**r**) the surface curvature. In our case, the QD formation in the grooves is driven by the surface curvature *κ*(**r**) which is accompanied by a high density of monolayer steps [25]. As shown in Fig. 1e, the curvature of the grooves does not depend on the pattern period, and thus, it does not explain the differences in the QD densities observed in Fig. 2. However, at high growth temperatures also, desorption should be taken into consideration [26, 27]. In our case the QDs were grown at 540 °C, which is high enough temperature to prevent QD formation on a planar sample but allows accumulation of In adatoms in the grooves. Therefore, the effect of the groove period on the size and density of the QDs can be attributed to a combined effect of migration and desorption of In atoms. The adatoms have a certain migration length on the surface until they are thermally desorbed. If the indium atoms reach a groove during that diffusion time, they will be incorporated into QDs. We observe the highest area density for the pattern with 200-nm groove period, where the grooves are very close together and no flat area exists between them, as shown in Fig. 1e. The QD area density for the 500-nm separated grooves is only slightly less than for the 200-nm separated grooves. In contrast to the overall QD area density, the linear QD density along the groove drastically increases (Fig. 2b) along with the QD height (Fig. 2c) when the groove period is increased from 200 to 500 nm. This can be explained by a reduction of favorable nucleation sites due to the increase of the groove separation. Desorption processes do not play a major role yet, and the amount of indium per groove is governed by the competition between the neighboring grooves over the adatoms. As the period is further increased to 1000 nm, the overall QD area density is further reduced, while the linear QD density slightly increases and the QD size increases from 11 to 15 nm. Beyond that point, both linear QD density and QD height saturate, and a difference is observed between the 1000- and 2000-nm separated grooves. In the saturation regime, there is no competition of In adatoms between the neighboring grooves because the separation between the grooves becomes larger than the migration length. Any adatoms arriving on the planar area too far away from a groove will desorb. In this regime, the overall area density of the QDs is inversely proportional to the groove period, as shown in Fig. 2a.

**Fig. 3:**
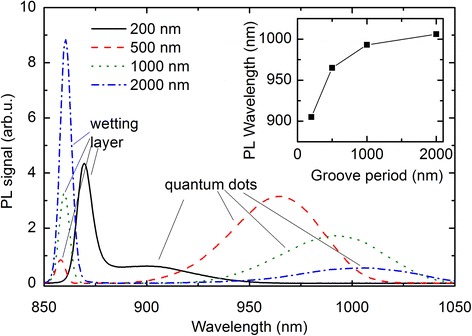
(color online) Ensemble PL measurements of QD chains with different periods as indicated in the figure. The *inset* shows the QD PL emission peak wavelength versus the groove period. The PL spectra were recorded at 20 K using an excitation power of 0.5 mW

In order to analyze the effects of the groove periods on the optical properties, in Fig. 3 we summarize the ensemble PL measurements. Below 870 nm wavelength, the emission from the wetting layers (as indicated in the figure) is observed. The QD PL emission is observed above 900 nm as broad peaks without any substructures. The peak wavelength of the QD emission is depicted in the inset of Fig. 3. With increasing groove period, the PL emission of the QDs is redshifted and starts to saturate above 1000 nm. Since the ground-state PL of QDs is mainly depending on the vertical confinement induced by the height of the QDs in growth direction, the PL emission is in agreement with AFM results showing an increasing, and subsequently saturating, QD height with larger groove periods.

**Fig. 4:**
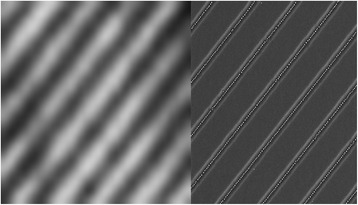
μ-PL image (*left*) and SEM image (*right*) of QD chains with a period of 1 μm, obtained from the PL and surface sample, respectively. The chains are aligned in [010] direction

Single QDC PL emission is demonstrated in Fig. 4 which shows on the left side a μ-PL image and on the right side a SEM picture of the corresponding surface sample. Intensity images were obtained using a diverging lens in order to increase the diameter of the laser spot on the sample to 30 μm and by measuring within the spectral range of 875–1000 nm using a high-pass filter in order to cut off the emission from the wetting layer and the substrate. We clearly observe PL emission from single QDCs as bright stripes. Because almost no QDs were observed between the grooves (see corresponding SEM image in Fig. 4 right), the PL emission of the QDCs is well separated.

**Fig. 5:**
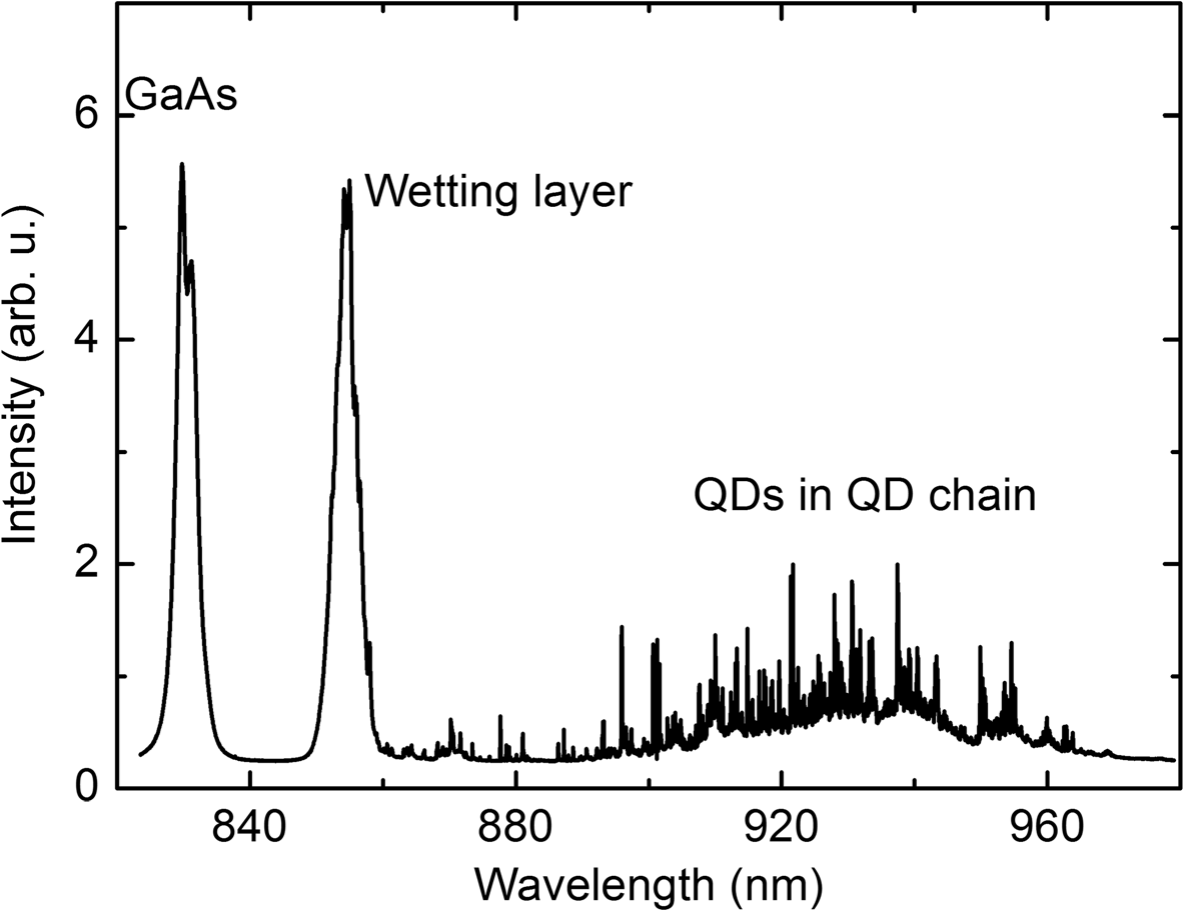
μ-PL spectra of a QD chain at 5 K

Spectral μ-PL measurements are shown in Fig. 5. QD emission is observed as sharp excitonic lines above a wavelength of 860 nm showing the opportunity to address the single QDs in the chain [28]. Furthermore, the PL emission of the wetting layer and GaAs bulk is visible as indicated in the figure.

## Conclusions

Structural and optical properties of site-controlled InAs QD chains have been studied depending on their groove period. Larger groove periods decrease the overall QD densities but increase the QD sizes and the linear QD densities in the groove. This can be explained by a combined effect of migration and desorption of indium atoms on the patterned and planar areas. The effect of the increased QD size with increased periods is reflected in ensemble PL measurements as redshift of the QD emission. Furthermore, single QD chain PL is demonstrated.

## Abbreviations

AFM: atomic force microscopy
Fig: figure
GaAs: gallium arsenide
InAs: indium arsenide
MBE: molecular beam epitaxy
NA: numerical aperture
PL: photoluminescence
PMT: photomultiplier tube
QD: quantum dot
QDC: quantum dot chain
SEM: scanning electron microscopy
UV-NIL: ultraviolet nanoimprint lithography
μ-PL: micro-photoluminescence
